# Knowledge and attitude toward postoperative antithrombotic management and prevention in patients with coronary revascularization: a cross-sectional study

**DOI:** 10.3389/fcvm.2024.1388164

**Published:** 2024-05-17

**Authors:** Chunlu Liu, Haijun Zhang, Liming Yang, Lihua Chen, Changhao Zu, Fangfang Wang, Yunjia Dai, Haiyan Zhao

**Affiliations:** ^1^ Department of Cardiology, Kailuan General Hospital, Tangshan, China; ^2^ Department of Cardiology, Tangshan Gongren Hospital, Tangshan, China

**Keywords:** myocardial infarction, knowledge, attitude, antiplatelet drugs, cross-sectional study

## Abstract

**Background:**

This study aimed to explore the knowledge and attitude (KA) toward postoperative antithrombotic management and prevention among coronary artery disease (CAD) patients who underwent coronary revascularization.

**Methods:**

This cross-sectional study enrolled CAD outpatients and inpatients between May and December 2023 at Kailuan Medical Group at Tangshan. Basic demographic characteristics and KA scores were collected through a self-made questionnaire.

**Results:**

This study included 523 valid questionnaires. The mean knowledge and attitude scores were 13.20 ± 6.20 (range: 0–26) and 43.68 ± 6.01 (range: 21–50), respectively, indicating poor knowledge and favorable attitude. Multivariable logistic regression analysis showed that junior high school education (OR = 2.160, *P* = 0.035), high school or technical school education (OR = 2.356, *P* = 0.039), and monthly average income >5,000 RMB (OR = 3.407, *P* = 0.002) were independently associated with knowledge. Knowledge (OR = 1.095, *P* = 0.002), BMI ≥ 24.0 kg/m^2^ (OR = 0.372, *P* = 0.011), junior high school (OR = 3.699, *P* = 0.002), high school or technical school (OR = 2.903, *P* = 0.028), high associate degree or above education (OR = 6.068, *P* = 0.014), monthly average income 3,000–5,000 RMB (OR = 0.296, *P* = 0.005), monthly average income > 5,000 RMB (OR = 0.225, *P* = 0.021), with hypertension (OR = 0.333, *P* = 0.003), blood tests every 2–3 weeks (OR = 10.811, *P* = 0.011), blood tests every month (OR = 4.221, *P* = 0.024), and blood tests every 2–3 months (OR = 3.342, *P* = 0.033) were independently associated with attitude.

**Conclusion:**

CAD patients who underwent coronary revascularization had poor knowledge but favorable attitudes toward postoperative antithrombotic management and prevention. The study underscores the need for targeted education, especially for individuals with lower education and income levels, ultimately improving patient compliance and cardiovascular outcomes.

## Background

Acute coronary syndromes (ACS) includes a spectrum of conditions associated with acute myocardial ischemia and/or necrosis, usually secondary to a reduction in coronary blood flow, including unstable angina (UA), non-ST-elevation myocardial infarction (NSTEMI), and ST-elevation myocardial infarction (STEMI) (1). The most common cause of ACS is plaque rupture in the setting of underlying coronary artery disease (CAD) (2). The common risk factors for CAD include tobacco abuse, dyslipidemia, hypertension, diabetes mellitus, and a family history of CAD (3). Revascularization of CAD can be performed electively or urgently when stenosis is significant (>50% stenosis) (4–7), and the most common type of surgery includes percutaneous coronary intervention (PCI) and coronary artery bypass grafting (CABG). CABG is a cardiac surgery in which a blood vessel segment, an artery or vein, is grafted from the aorta to bypass a blockage in a coronary artery, creating a new conduit to enhance blood flow to the heart (8), while PCI involves balloon dilation and stent implantation (9), and they both improve patients' survival outcome (10).

Antiplatelet therapy is recommended after CABG or PCI and is an important factor in improving patients' outcomes. Indeed, antiplatelet therapy decreases the risk of vein graft failure and major adverse cardiovascular events (MACE) (11) but at the expense of an increased bleeding risk (12). The antiplatelet therapies are mainly oral drugs that are taken at home without supervision. Hence, compliance with treatment is an important factor affecting the prognosis of coronary revascularization. Compliance with guideline-directed medical therapy following CABG or PCI was reported to be 67% at 1 year and 53% at 5 years (13). It is worth emphasizing that patient adherence to treatment is crucial and depends on the individual's understanding of medication purposes, recognizing warning signs, being aware of potential side effects, and having a positive attitude toward the treatment (14, 15). Postoperative antithrombotic therapy plays a crucial role in preventing thrombotic events and ensuring successful outcomes in patients undergoing coronary revascularization. Patient knowledge and attitude toward antithrombotic management are essential factors influencing treatment adherence and effectiveness. However, existing literature lacks a comprehensive understanding of the specific gaps in patient knowledge and attitude regarding postoperative antithrombotic therapy, particularly in the context of coronary revascularization.

Hence, evaluating the knowledge and attitude (KA) of antiplatelet therapy after coronary revascularization could provide important data about the gaps, misconceptions, and misunderstandings that could constitute barriers to optimal treatments (16, 17). Although several studies reported viewpoints of healthcare providers on antiplatelet therapy (18, 19), there were no related studies discussing KA toward postoperative antithrombotic management and prevention in patients with coronary revascularization. Assessing knowledge and attitude (KA) could allow the design of educational and motivational interventions to improve the practice of patients with CAD toward antiplatelet drugs, possibly improving patient outcomes. Therefore, this study aimed to investigate the KA toward postoperative antiplatelet management and prevention among CAD patients after coronary revascularization.

## Methods

### Study design and participants

This cross-sectional study enrolled CAD outpatients and inpatients between May and December 2023 at Kailuan Medical Group in Tangshan, Hebei Province, China. The inclusion criteria were: (1) clinically diagnosed with CAD or underwent PCI or CABG due to CAD, and (2) were well-informed about the study and voluntarily participated in this study. The exclusion criteria were: (1) consciousness, mental, or cognitive impairments, (2) malignant tumors, (3) severe systemic infectious diseases, or (4) immune system disorders. The research protocol was approved by the ethics committee of Kailuan General Hospital. Informed consents were obtained from the participants before they completed the survey. This study is reported according to the CROSS statement (20).

### Questionnaire

The questionnaire design was based on the 2021 ACC/AHA/SCAI Guidelines for Coronary Revascularization (11) and the relevant literature (1, 10, 12, 13, 21). After the initial design, feedback from three experts, including two specialists in cardiovascular medicine and one in epidemiology, was sought and used to refine the questionnaire. Forty individuals were enrolled for a pretest to assess the reliability and validity of the questionnaire. The pretest reliability analysis showed that the Cronbach's *α* coefficients for knowledge, attitude, and overall were 0.892, 0.621, and 0.865, respectively, indicating good internal consistency. The overall data analysis showed that the Cronbach's *α* coefficients for knowledge, attitude, and overall were 0.838, 0.953, and 0.886, indicating good internal consistency. The Kaiser-Meyer-Olkin (KMO) measure was 0.924, suggesting good validity.

The final questionnaire was in Chinese and encompassed three parts: basic demographic characteristics, knowledge dimension, and attitude dimension. The basic demographic characteristics section comprised 14 questions on demographic and socioeconomic characteristics. The knowledge dimension consisted of 14 questions, with responses categorized into “Aware”, “Moderately aware”, and “Unaware”, scored 2, 1, and 0 points, respectively. The total score range was from 0 to 26 points. The 14th question investigated the sources of knowledge about antithrombotic management, without assigning scores. The attitude dimension comprised 10 questions and employed a 5-point Likert scale. For positively framed questions, responses ranged from “Strongly agree” to “Strongly disagree” scored from 5 to 1. Conversely, negatively framed questions are scored in reverse. The attitude score range was 10–50 points. Knowledge was deemed sufficient, and attitude was considered positive if the score exceeded 75% of the total score in the attitude dimension (22).

### Questionnaire distribution and quality control

The survey questionnaires were distributed to the study participants through two channels: WeChat groups and face-to-face interactions in clinical examination rooms. The WeChat groups were created by the clinic to advertise medical information and news to patients. The study was simply advertised, and those interested in participating could participate. The patients have also proposed the study during routine medical examinations. Trained research team members checked all questionnaires for validity. Incomplete questionnaires, the questionnaires that took <30 s or >480 s to complete, had obvious logical errors [e.g., impossible age or body mass index (BMI, kg/m^2^)] or were completed using all the same options (e.g., all first choices) and were considered invalid.

### Sample size calculation

The sample size was calculated using the formula for cross-sectional studies: α=0.05, $n\;=\;[Z(1-\alpha /2)/\delta {]}^{2}\;\times \;p\;\;\times \;\;(1-p)\;$ whereZ_(1−α/2)=1.96 whenα=0.05, the assumed degree of variability of *p* = 0.5 maximizes the required sample size, and *δ* is an admissible error (which was 5% here). The theoretical sample size was 480, which included an extra 20% to allow for subjects to be lost during the study.

### Statistical analysis

Data analysis was performed using SPSS 22.0 (IBM, Armonk, NY, USA). They were tested for normal distribution using the Kolmogorov-Smirnov test. Continuous data with a normal distribution were described as means ± standard deviation (SD) and analyzed using Student's t-test; otherwise, they were presented as medians (interquartile range, IQR) and analyzed using the Wilcoxon rank-sum test. Univariable and multivariable logistic regression analyses were used to analyze the covariates independently associated with knowledge and attitude. Variables with *P* < 0.05 in the univariable analyses were included in the multivariable analyses (enter method). A two-sided *P*-value < 0.05 was considered statistically significant.

## Results

### Basic characteristics of the participants

Eleven questionnaires with an impossible BMI, one questionnaire from a participant reportedly under the age of 18, and two questionnaires with all responses in the knowledge and attitude dimension marked as “A” were excluded. Hence, 523 valid questionnaires were included in the analysis (validity rate of 97.39%). Most participants were >70 years old (30.02%), overweight (61.95%), male (68.07%), were urban residence (55.83%), with junior high school education (43.21%), earning monthly average income of 3,000–5,000 RMB (52.77%), not smoking (58.89%), not drinking (64.05%), not consuming coffee or tea (86.81%), with hypertension (69.98%), without diabetes (60.23%), without hyperlipidemia (78.01%), without a history of coronary heart disease (79.92%), >5 years since ACS (34.80%), underwent PCI (95.79%), taking antithrombotic medication (84.51%), and undergoing blood tests every 2–3 months (58.70%) (Table 1).

**Table 1:** Basic characteristics, knowledge and attitude scores.

Variable	*n* (%)	Knowledge		Attitude	
** **		Mean ± SD	*P*	Mean ± SD	*p*
Total	523	13.20 ± 6.20		43.68 ± 6.01	
Age (years)			0.566		0.973
<60	147 (28.11)	13.59 ± 6.24		43.59 ± 5.97	
60–65	104 (19.89)	13.61 ± 6.51		43.70 ± 5.91	
66–70	115 (21.99)	12.74 ± 6.04		43.56 ± 5.96	
>70	157 (30.02)	12.89 ± 6.09		43.86 ± 6.20	
Body mass index (kg/m^2^)			0.401		0.021
<24.0	199 (38.05)	12.90 ± 5.93		44.46 ± 5.34	
≥24.0	324 (61.95)	13.37 ± 6.36		43.21 ± 6.35	
Gender			0.679		0.448
Male	356 (68.07)	13.12 ± 6.21		43.55 ± 6.11	
Female	167 (31.93)	13.36 ± 6.19		43.98 ± 5.81	
Residence			0.293		0.023
Urban	292 (55.83)	13.45 ± 6.40		43.15 ± 6.13	
Rural	231 (44.17)	12.87 ± 5.93		44.35 ± 5.81	
Education			<0.001		0.149
Primary school or below	126 (24.09)	11.35 ± 5.68		42.61 ± 6.59	
Junior high School	226 (43.21)	13.27 ± 6.20		44.04 ± 5.71	
High school/technical school	115 (21.99)	13.98 ± 6.10		43.95 ± 5.97	
Associate degree and above	56 (10.71)	15.45 ± 6.53		44.13 ± 5.80	
Monthly average income (RMB)			<0.001		0.467
<3,000	175 (33.46)	11.83 ± 5.82		44.04 ± 5.51	
3,000–5,000	276 (52.77)	12.82 ± 5.90		43.63 ± 6.44	
>5,000	72 (13.77)	17.94 ± 6.06		43.01 ± 5.46	
Smoking			0.898		0.963
Yes	215 (41.11)	13.15 ± 6.14		43.67 ± 6.29	
No	308 (58.89)	13.22 ± 6.25		43.69 ± 5.82	
Drinking			0.400		0.713
Yes	188 (35.95)	13.50 ± 6.40		43.81 ± 6.27	
No	335 (64.05)	13.02 ± 6.08		43.61 ± 5.87	
Regular consumption of coffee or tea			0.042		<0.001
Yes	69 (13.19)	14.61 ± 6.54		40.68 ± 5.47	
No	454 (86.81)	12.98 ± 6.12		44.14 ± 5.97	
Hypertension			0.410		0.213
Yes	366 (69.98)	13.34 ± 6.12		43.90 ± 6.22	
No	157 (30.02)	12.85 ± 6.38		43.18 ± 5.49	
Diabetes			0.164		0.663
Yes	208 (39.77)	12.73 ± 5.86		43.54 ± 6.41	
No	315 (60.23)	13.50 ± 6.41		43.78 ± 5.74	
Hyperlipidemia			0.021		<0.001
Yes	115 (21.99)	12.02 ± 6.27		39.30 ± 5.36	
No	408 (78.01)	13.53 ± 6.15		44.92 ± 5.60	
Family history of coronary heart disease			0.167		<0.001
Yes	105 (20.08)	13.94 ± 6.58		40.31 ± 6.05	
No	418 (79.92)	13.01 ± 6.09		44.53 ± 5.70	
Duration of coronary heart disease affliction			0.148		0.839
<1 year	177 (33.84)	12.46 ± 6.54		43.89 ± 6.16	
1–5 years	164 (31.36)	13.63 ± 6.31		43.51 ± 6.01	
>5 years	182 (34.80)	13.52 ± 5.71		43.65 ± 5.89	
Type of coronary artery revascularization procedure			0.964		0.147
Percutaneous coronary intervention (PCI)	501 (95.79)	13.20 ± 6.14		43.76 ± 5.94	
Coronary artery bypass grafting (CABG)	22 (4.21)	13.14 ± 7.50		41.86 ± 7.45	
Currently taking antithrombotic medications			<0.001		0.002
Yes	442 (84.51)	13.66 ± 6.15		44.03 ± 6.17	
No	81 (15.49)	10.64 ± 5.89		41.80 ± 4.64	
Frequency of routine blood tests while taking antithrombotic medication			0.083		0.002
Weekly	26 (4.97)	15.23 ± 6.45		40.92 ± 6.69	
Every 2–3 weeks	47 (8.99)	14.13 ± 5.40		44.87 ± 5.48	
Monthly	143 (27.34)	13.62 ± 6.79		44.84 ± 6.10	
Every 2–3 months	307 (58.70)	12.68 ± 5.96		43.20 ± 5.87	

### Knowledge dimension

The mean knowledge score was 13.20 ± 6.20 (range: 0–26), indicating poor knowledge. Higher knowledge scores were observed with higher education (*P* < 0.001), higher income (*P* < 0.001), consumption of coffee or tea (*P* = 0.042), those without hyperlipidemia (*P* = 0.021), and currently taking antithrombotic medication (*P* < 0.001) (Table 1). The items with the poorest knowledge scores were K9 [25.43%; “Novel oral anticoagulants (NOACs), mainly including apixaban, rivaroxaban, and dabigatran”] and K8 (31.40%; “Warfarin is the most commonly used anticoagulant therapy drug”). Hospital lectures and education were the primary sources of knowledge (47.42%) (Table 2).

**Table 2:** Knowledge dimension.

Knowledge, *n* (%)	Aware	Moderately aware			Unaware
K1. Coronary artery disease is a type of heart artery disease, and coronary heart disease is the most common type	244 (46.65)	159 (30.40)			120 (22.94)
K2. Modifiable risk factors for cardiovascular disease include blood pressure, cholesterol, smoking, obesity, etc.	297 (56.79)	161 (30.78)			65 (12.43)
K3. Coronary artery revascularization procedures include percutaneous coronary intervention (PCI) and coronary artery bypass grafting (CABG)	209 (39.96)	150 (28.68)			164 (31.36)
K4. Major complications after coronary artery revascularization surgery include in-stent thrombosis, low blood pressure, and arrhythmia	168 (32.12)	122 (23.33)			233 (44.55)
K5. Antithrombotic treatment is divided into antiplatelet therapy and anticoagulant therapy	112 (21.41)	100 (19.12)			311 (59.46)
K6. Antithrombotic treatment after coronary artery surgery should be maintained for life	262 (50.10)	218 (41.68)			43 (8.22)
K7. Aspirin is the most commonly used antiplatelet therapy drug	273 (52.20)	226 (43.21)			24 (4.59)
K8. Warfarin is the most commonly used anticoagulant therapy drug	80 (15.30)	79 (15.11)			364 (69.60)
K9. Novel oral anticoagulants (NOACs), mainly including apixaban, rivaroxaban, and dabigatran	65 (12.43)	68 (13.00)			390 (74.57)
K10. Routine blood tests, fecal occult blood, and coagulation function should be regularly checked during antithrombotic treatment	220 (42.07)	217 (41.49)			86 (16.44)
K11. The main complication of antithrombotic treatment is bleeding.	228 (43.59)	238 (45.51)			57 (10.90)
K12. Antithrombotic treatment should be used cautiously in case of severe liver and kidney dysfunction, poor coagulation function, etc.	127 (24.28)	85 (16.25)			311 (59.46)
K13. Percutaneous coronary intervention (PCI) is one of the thrombolytic treatment methods	197 (37.67)	114 (21.80)			212 (40.54)
	Medical-related books and materials	Hospital lectures and education	New media	Multi-media	Relatives and friends
K14. Where do you generally acquire knowledge about antithrombotic management?	48 (9.18)	248 (47.42)	91 (17.40)	38 (7.27)	98 (18.74)

### Attitude dimension

The mean attitude score was 43.68 ± 6.01 (range: 21–50), indicating a favorable attitude. Higher attitude scores were observed with a smaller BMI (*P* = 0.021), rural residence (*P* = 0.023), no consumption of coffee or tea (*P* < 0.001), those without hyperlipidemia (*P* = 0.021), those with a family history of CAD (*P* < 0.001), currently taking antithrombotic medication (*P* = 0.002), and undergoing blood tests every 2–4 weeks (*P* = 0.002) (Table 1). Table 3 presents the distribution of the attitude of each item.

**Table 3:** Attitude dimension.

Attitude, *n* (%)	Strongly agree	Agree	Neutral	Disagree	Strongly disagree
A1. I worry about the occurrence of thrombosis after surgery	250 (47.80)	212 (40.54)	44 (8.41)	16 (3.06)	1 (0.19)
A2. I worry that thrombosis after surgery will threaten my life	260 (49.71)	215 (41.11)	31 (5.93)	17 (3.25)	0
A3. I believe that antithrombotic treatment is necessary after surgery	241 (46.08)	228 (43.59)	49 (9.37)	5 (0.96)	0
A4. I believe that implementing effective preventive measures will improve postoperative quality of life	253 (48.37)	233 (44.55)	30 (5.74)	6 (1.15)	1 (0.19)
A5. I worry that there will be complications with antithrombotic treatment after surgery	247 (47.23)	219 (41.87)	39 (7.45)	17 (3.25)	1 (0.19)
A6. I believe that regardless of preoperative or postoperative, quitting smoking is necessary to reduce the risk of thrombosis	270 (51.63)	219 (41.87)	26 (4.97)	8 (1.53)	0
A7. I believe that in addition to antithrombotic drug treatment, it is necessary to coordinate with glycemic, lipid-lowering, and antihypertensive treatments according to the condition	257 (49.14)	234 (44.74)	26 (4.97)	6 (1.15)	0
A8. I believe that long-term antithrombotic treatment increases my financial burden	227 (43.40)	196 (37.48)	73 (13.96)	27 (5.16)	0
A9. I believe that when facing long-term antithrombotic treatment, family support is very important	261 (49.90)	224 (42.83)	26 (4.97)	10 (1.91)	2 (0.38)
A10. I believe that hospitals can regularly hold lectures on antithrombotic treatment	281 (53.73)	210 (40.15)	27 (5.16)	5 (0.96)	0

### Multivariable logistic regression analysis

Multivariable logistic regression analysis found that junior high school (OR = 2.160, 95% CI: 1.058–4.411, *P* = 0.035), high school or technical school (OR = 2.356, 95% CI: 1.045–5.312, *P* = 0.039), and monthly average income >5,000 RMB (OR = 3.407, 95% CI: 1.619–7.166, *P* = 0.002) were independently associated with knowledge (Table 4). The knowledge (OR = 1.095, 95% CI: 1.032–1.160, *P* = 0.002), BMI ≥ 24.0 kg/m^2^ (OR = 0.372, 95% CI: 0.175–0.794, *P* = 0.011), junior high school (OR = 3.699, 95% CI: 1.615–8.473, *P* = 0.002), high school or technical school (OR = 2.903, 95% CI: 1.124–7.500, *P* = 0.028), high associate degree or above (OR = 6.068, 95% CI: 1.449–25.408, *P* = 0.014), monthly average income 3,000–5,000 RMB (OR = 0.296, 95% CI: 0.126–0.692, *P* = 0.005), monthly average income >5,000 RMB (OR = 0.225, 95% CI: 0.063–0.799, *P* = 0.021), with hypertension (OR = 0.333, 95% CI: 0.160–0.693, *P* = 0.003), blood tests every 2–3 weeks (OR = 10.811, 95% CI: 1.732–67.487, *P* = 0.011), blood tests every month (OR = 4.221, 95% CI: 1.213–14.691, *P* = 0.024), and blood tests every 2–3 months (OR = 3.342, 95% CI: 1.105–10.109, *P* = 0.033) were independently associated with attitude (Table 5).

**Table 4:** Univariable and multivariable logistic regression analysis of knowledge.

	Univariable analysis		Multivariable analysis	
	OR (95%CI)	*P*	OR (95%CI)	*P*
Age (years)				
<60	Ref.			
60–65	1.046 (0.564–1.942)	0.886		
66–70	0.772 (0.409–1.456)	0.424		
>70	0.960 (0.547–1.682)	0.885		
Body mass index (kg/m^2^)				
<24.0	Ref.			
≥24.0	1.158 (0.708–1.818)	0.523		
Gender				
Male	Ref.			
Female	0.864 (0.539–1.385)	0.543		
Residence				
Urban	Ref.			
Rural	0.738 (0.474–1.150)	0.180		
Education				
Primary school or below	Ref.		Ref.	
Junior high School	2.362 (1.198–4.655)	0.013	2.160 (1.058–4.411)	0.035
High school/technical school	3.057 (1.471–6.354)	0.003	2.356 (1.045–5.312)	0.039
Associate degree and above	4.141 (1.817–9.436)	0.001	2.215 (0.863–5.688)	0.098
Monthly average income (RMB)				
<3,000	Ref.		Ref.	
3,000–5,000	1.391 (0.813–2.382)	0.229	1.085 (0.603–1.929)	0.785
>5,000	4.997 (2.634–9.479)	< 0.001	3.407 (1.619–7.166)	0.002
Smoking				
Yes	0.954 (0.614–1.483)	0.835		
No	Ref.			
Drinking				
Yes	1.253 (0.804–1.952)	0.319		
No	Ref.			
Regular consumption of coffee or tea				
Yes	2.015 (1.144–3.549)	0.015	1.318 (0.697–2.492)	0.396
No	Ref.		Ref.	
Hypertension				
Yes	1.292 (0.770–2.167)	0.333		
No	Ref.			
Diabetes				
Yes	1.168 (0.721–1.890)	0.528		
No	Ref.			
Hyperlipidemia				
Yes	0.602 (0.378–0.957)	0.032	0.653 (0.399–1.067)	0.089
No	Ref.		Ref.	
Family history of coronary heart disease				
Yes	0.902 (0.530–1.535)	0.704		
No	Ref.			
Duration of coronary heart disease affliction				
<1 year	Ref.			
1–5 years	1.274 (0.748–2.170)	0.372		
>5 years	1.041 (0.610–1.776)	0.883		
Type of coronary artery revascularization procedure				
Percutaneous coronary intervention (PCI)	Ref.			
Coronary artery bypass grafting (CABG)	1.225 (0.441–3.402)	0.697		
Currently taking antithrombotic medications				
Yes	1.650 (0.839–3.244)	0.147		
No	Ref.			
Frequency of routine blood tests while taking antithrombotic medication				
Weekly	Ref.		Ref.	
Every 2–3 weeks	0.387 (0.128–1.175)	0.094	0.465 (0.141–1.529)	0.135
Monthly	0.589 (0.241–1.442)	0.247	0.683 (0.257–1.815)	0.445
Every 2–3 months	0.376 (0.159–0.891)	0.026	0.404 (0.157–1.041)	0.061

**Table 5:** Univariable and multivariable logistic regression analysis of attitude.

	Univariable analysis		Multivariable analysis	
	OR (95%CI)	*P*	OR (95%CI)	*P*
Knowledge score	1.081 (1.026–1.140)	0.004	1.095 (1.032–1.160)	0.002
Age (years)				
<60	Ref.			
60–65	1.148 (0.499–2.642)	0.745		
66–70	1.439 (0.611–3.385)	0.405		
>70	1.076 (0.517–2.239)	0.844		
Body mass index (kg/m^2^)				
<24.0	Ref.		Ref.	
≥24.0	0.415 (0.208–0.830)	0.013	0.372 (0.175–0.794)	0.011
Gender				
Male	Ref.			
Female	1.140 (0.606–2.146)	0.685		
Residence				
Urban	Ref.			
Rural	1.659 (0.902–3.052)	0.104		
Education				
Primary school or below	Ref.		Ref.	
Junior high school	2.049 (1.015–4.136)	0.045	3.699 (1.615–8.473)	0.002
High school/technical school	1.431 (0.657–3.117)	0.367	2.903 (1.124–7.500)	0.028
Associate degree and above	2.167 (0.698–6.726)	0.181	6.068 (1.449–25.408)	0.014
Monthly average income (RMB)				
<3,000	Ref.		Ref.	
3,000–5,000	0.431 (0.207–0.897)	0.024	0.296 (0.126–0.692)	0.005
>5,000	0.563 (0.205–1.541)	0.263	0.225 (0.063–0.799)	0.021
Smoking				
Yes	1.313 (0.719–2.400)	0.375		
No	Ref.			
Drinking				
Yes	1.136 (0.616–2.095)	0.682		
No	Ref.			
Regular consumption of coffee or tea				
Yes	0.509 (0.248–1.048)	0.067		
No	Ref.			
Hypertension				
Yes	0.309 (0.169–0.567)	<0.001	0.333 (0.160–0.693)	0.003
No	Ref.		Ref.	
Diabetes				
Yes	0.871 (0.456–1.660)	0.674		
No	Ref.			
Hyperlipidemia				
Yes	0.857 (0.478–1.537)	0.605		
No	Ref.			
Family history of coronary heart disease				
Yes	0.355 (0.195–0.648)	0.001	0.669 (0.325–1.377)	0.275
No	Ref.		Ref.	
Duration of coronary heart disease affliction				
<1 year	Ref.			
1–5 years	0.983 (0.479–2.016)	0.962		
>5 years	0.968 (0.482–1.945)	0.927		
Type of coronary artery revascularization procedure				
Percutaneous coronary intervention (PCI)	Ref.		Ref.	
Coronary artery bypass grafting (CABG)	0.344 (0.121–0.975)	0.045	0.408 (0.119–1.399)	0.154
Currently taking antithrombotic medications				
Yes	0.706 (0.291–1.713)	0.441		
No	Ref.			
Frequency of routine blood tests while taking antithrombotic medication				
Weekly	Ref.		Ref.	
Every 2–3 weeks	8.289 (1.575–43.616)	0.013	10.811 (1.732–67.487)	0.011
Monthly	5.485 (1.829–16.452)	0.002	4.221 (1.213–14.691)	0.024
Every 2–3 months	3.059 (1.196–7.822)	0.020	3.342 (1.105–10.109)	0.033

## Discussion

This study found that patients with CAD who underwent coronary revascularization have poor knowledge but favorable attitudes toward postoperative antithrombotic management and prevention. The questionnaire covered several aspects of antithrombotic management, but the patterns of poor knowledge were different among the participants. In addition, categories of patients were identified as associated with poorer knowledge, underscoring the importance of tailored patient education programs aimed at improving knowledge levels, particularly among individuals with lower education and income.

In this study, most patients with CAD were above 60 years old, which is consistent with the epidemiology of ACS (1, 2, 10, 23). The participants in the present study were older than in two previous KAP studies in patients with CAD (24, 25), suggesting a small likelihood of selection bias in the present study. The smoking rate in this study was lower than that in the other two previous studies (24, 26). Two previous studies reported that 97% (24) and 71% (25) of the participants could name the symptoms of CAD. Akshay et al. (24) reported that 50% of participants knew what CAD is, and 66.1% knew that the patients should follow a specific diet. Mohammad et al. (27) reported that about 50% of their participants could correctly answer general CAD questions. Similar results were observed in the present study. The questionnaire covered knowledge areas like the nature of CAD, the risk factors for CAD, the management of CAD, complications after CAD surgery, antithrombotic types, the length of antithrombotic therapy, the antithrombotic drugs available, examinations to perform during antithrombotic treatment, and the complications of antithrombotic treatment. Still, the patterns of incorrect responses were different among participants. A future study should examine whether the questionnaire developed here could be used to identify the KA areas to be improved and provide tailored educational and motivational interventions to the patients.

In the present study, higher education and higher income were both associated with higher knowledge scores. It is well known that a more favorable socioeconomic status is associated with better health literacy (28). Similar results were also observed previously (24, 29). Higher BMI was associated with lower attitudes, possibly because they tended to have poorer lifestyle habits and low willingness to change them. Higher education was also associated with a more favorable attitude. Surprisingly, higher income was associated with a less favorable attitude. It could be because patients with lower incomes are more willing to cultivate a good attitude toward therapy to avoid MACE, complications, and additional medical expenses. Less frequent blood examinations were also associated with a more favorable attitude, possibly because of saved time and money. Hence, these results suggest that although all patients would benefit from educational interventions, those with a lower socioeconomic status could profit even more.

This study also found that knowledge was independently associated with attitude, indicating that improving knowledge through education should also improve the attitude. This study showed that all patients would benefit from education, but those with low education and low income, i.e., those with lower socioeconomic status, would benefit the most. Still, available data in the literature suggested that healthcare providers have moderate knowledge of antiplatelet therapy (18, 19). Considering that healthcare providers are a primary source of reliable health information, as in the present study, their knowledge of antiplatelet therapy should be improved.

However, this study still had several limitations. The study was performed at a single center, resulting in a relatively small sample size. In addition, the participants were all from the same geographical area, limiting the generalizability of the results to other areas. The questionnaire was designed by the investigators according to the literature and local practice, policies, and reality. Hence, the questionnaire has limited exportability, and the results could lack generalizability. The items about the clinical characteristics of the participants were self-reported, and the results could be biased. In the present study, the practice was not evaluated because compliance, as the main practice indicator, could not be determined reliably. Finally, all KAP studies are at risk of the social desirability bias (30, 31), but considering that knowledge was poor, that bias has a low likelihood.

In conclusion, this study underscored the importance of tailored patient education programs aimed at improving knowledge levels, particularly among individuals with lower education and income. Addressing these gaps in understanding may enhance overall patient compliance and contribute to better cardiovascular outcomes following coronary revascularization. Future interventions should consider these factors to optimize postoperative antithrombotic management and prevention strategies among CAD patients.

## Data Availability

The original contributions presented in the study are included in the article/Supplementary Material, further inquiries can be directed to the corresponding author.
